# RETRACTED: Qu et al. Kinetics of Martensite/Austenite Decomposition during Tempering of Ultrafine Nano-Bainitic Steels. *Materials* 2024, *17*, 2690

**DOI:** 10.3390/ma18184265

**Published:** 2025-09-12

**Authors:** Zhiwei Qu, Min Lei, Guohua Chen, Chaowen Huang, Dan Liu, Ai Luo

**Affiliations:** 1 College of Materials and Metallurgy, Guizhou University, Guiyang 550025, China; quzhiwei1999@163.com (Z.Q.); 16685317895@163.com (G.C.); cwhuang@gzu.edu.cn (C.H.); liud@gzu.edu.cn (D.L.); 18716634254@163.com (A.L.); 2 Key Laboratory for Materials Structure and Strength of Guizhou Province, Guiyang 550025, China

The journal retracts the article titled “Kinetics of Martensite/Austenite Decomposition during Tempering of Ultrafine Nano-Bainitic Steels” [1], cited above.

Following publication, the authors contacted the Editorial Office to raise concerns regarding the scientific accuracy of the data presented in the published paper [1].

Adhering to our standard procedure, an investigation was conducted by the Editorial Office and the Editorial Board. Following discussion with the authors, the Editorial Board concluded that a range of flaws, such as issues with the Fourier Transform that impact the judgment of the tissue; residual stress and the limitations of the pdf card database; errors in the discussion in relating to Figure 10; and the overall deficiencies in the methods to establish the presence of M/A, seriously impacts the reliability of the findings of this study [1]. As a result, the Editorial Board has decided to retract this article [1] as per MDPI’s retraction policy (https://www.mdpi.com/ethics#_bookmark30).

This retraction was approved by the Editor-in-Chief of the *Materials* journal.

The authors agreed to this retraction.
